# A Systematic Review and Meta-Analysis of the Awareness of and Attitudes Toward Epistaxis

**DOI:** 10.7759/cureus.46590

**Published:** 2023-10-06

**Authors:** Khalid M Alkhalifah, Norah I Alhumaidan, Turki A Alotaibi, Rakan Fiasel M Almnjwami, Lama A Alzelfawi, Rasil H Almughamsi, Renad K Alqahtani, Mubarak H Aldossari, Yahya A Fageeh

**Affiliations:** 1 Unaizah College of Medicine and Medical Sciences, Qassim University, Unaizah, SAU; 2 College of Medicine, Princess Nourah bint Abdulrahman University, Riyadh, SAU; 3 College of Medicine, Taif University, Taif, SAU; 4 Department of ENT, Al-Hada Armed Forces Hospital, Taif, SAU; 5 College of Medicine, Taibah University, Medina, SAU; 6 College of Medicine, Imam Mohammad Ibn Saud Islamic University, Riyadh, SAU; 7 College of Medicine, Prince Sattam Bin Abdulaziz University, Al-Kharj, SAU; 8 Otolaryngology - Head and Neck Surgery, College of Medicine, Taif University, Taif, SAU

**Keywords:** attitude, knowledge, awareness, nosebleed, nasal bleeding, epistaxis

## Abstract

Epistaxis, commonly known as nasal bleeding, ranks among the most prevalent emergencies encountered in otorhinolaryngology. The etiology of epistaxis is multifaceted, arising from both local and systemic factors. In Saudi Arabia, a country with a relatively high prevalence of epistaxis, understanding the level of awareness and attitudes toward first aid management of epistaxis is of paramount importance. This systematic review aims to bridge this knowledge gap by evaluating the awareness of and attitudes toward epistaxis first aid in Saudi Arabia.

This systematic review and meta-analysis adhered to Preferred Reporting Items for Systematic Review and Meta-Analysis (PRISMA) guidelines. A comprehensive electronic search was executed across PubMed, Google Scholar, and Web of Science databases, encompassing studies published between January 2015 and July 2023. The study included exclusively cross-sectional studies, assessing awareness and attitude toward epistaxis first aid in Saudi Arabia across all populations and studies in English.

The 17 selected studies were all published after October 2017, with three published in the year of this systematic review (2023). Sample sizes exhibited substantial variability, ranging from 57 to 2,441 individuals. Despite widespread awareness of epistaxis, the general population often disregards it as a minor health issue. This discrepancy highlights the importance of addressing epistaxis seriously, given the potential for severe bleeding as a medical emergency. The review of 17 studies revealed significant variations in epistaxis awareness levels, influenced by factors such as age, gender, and varying sample sizes. Notably, higher awareness levels were observed in studies involving the general Saudi population and those employing self-administered questionnaires.

The average awareness and knowledge of epistaxis and its management among Saudi residents were moderate, with an estimated awareness level of 63%. A large-scale epidemiological survey, considering sociodemographic factors, is recommended to provide a more comprehensive understanding of epistaxis awareness.

## Introduction and background

Epistaxis, or nasal bleeding, is the most encountered emergency in otorhinolaryngology [1]. Epistaxis, whether spontaneous or otherwise, is experienced by up to 60% of people in their lifetime, with 6% requiring medical attention, accounting for one in 200 visits in the United States, affecting those less than 10 and older than 60 years of age [2]. Epistaxis appears to be more common in winter than in summer [3]. There are many causes for nasal bleeding, which can include local or systemic factors. The most frequent local factors include upper airway infections, trauma, cold and dry air-breathing, nasal allergies, the introduction of foreign bodies in the nasal cavity, septal perforation or deviation, and tumors. Among the systemic causes are elevated arterial blood pressure, blood disorders, coagulopathy, and the use of anticoagulants [4]. From a clinical perspective, epistaxis is classified as either anterior or posterior based on the anatomy of the blood supply of the nose [5]. Anterior epistaxis is more commonly observed during the early stages of life. It can originate from an arterial source, particularly the Kiesselbach's plexus, or venous sources, such as the retro-columellar vein. Due to the ease of accessing the bleeding point, this form of epistaxis tends to be less severe. In contrast, posterior bleeding derives primarily from the posterior septal nasal artery (a branch of the sphenopalatine artery), which forms part of the Woodruff plexus. It is more prevalent among older people and can pose significant therapeutic challenges [6]. Treatment options range from simple manual compression, cauterization, and packing to endoscopic ligation or embolization [4]. According to the available data, the prevalence of epistaxis is estimated to impact roughly 10%-12% of the population. Among these cases, approximately 10% are classified as severe and need specialized medical attention [7].

Several studies have explored the awareness and attitudes towards epistaxis and first aid measures in different populations. A survey conducted by Alotaibi et al. (2019) aimed to assess Saudi teachers' knowledge, practices, and attitudes toward epistaxis management in schools [8]. Another study by Alshehri et al. (2020) investigated the first aid knowledge and practices among parents in Saudi Arabia regarding epistaxis in children [9].

In Saudi Arabia, where the prevalence of epistaxis is relatively high, understanding the level of first aid awareness and the attitudes of individuals toward managing epistaxis is of utmost importance [10]. Assessing the knowledge and practices of the general population can provide valuable insights into the adequacy of first aid education, identify gaps in knowledge, and improve the overall management of epistaxis incidents. Therefore, this systematic review aims to assess awareness and attitude toward epistaxis first aid in Saudi Arabia.

## Review

Methods

Literature Search Strategy

This Systematic Review and Meta-Analysis was conducted in adherence to Preferred Reporting Items for Systematic Review and Meta-Analysis (PRISMA) guidelines. A broad electronic search was conducted through PubMed, Google Scholar, EBSCO, and Web of Science databases for studies published between January 2000 and July 2023. For the search strategy, these terms: (“Epistaxis” OR “Nasal Bleeding” OR “Nosebleed”) AND (“Awareness” OR “Knowledge “OR “attitude” OR “practice”) AND (“Saudi Arabia” OR “Kingdom of Saudi Arabia”) were used to identify all studies related to epistaxis first aid awareness and attitude in Saudi Arabia.

Inclusion and Exclusion Criteria

This systematic review included all cross-sectional studies that assessed awareness and attitude toward Epistaxis first aid in Saudi Arabia on all populations and in English. Exclusion criteria included all types of studies other than cross-sectional and studies conducted in other languages.

Selection of Articles and Data Extraction

All articles from the primary search were imported to Mendeley for duplication removal. The final result after deduplication was imported to Rayyan and independently screened by four authors (T.A.A., R.F.A., N.I.A., R.H.A.) based on title and abstract. One author reviewed the full text of all studies (N.I.A.). Disagreements at any step of the screening process were handled through debate and consensus among all authors.

**Figure 1:**
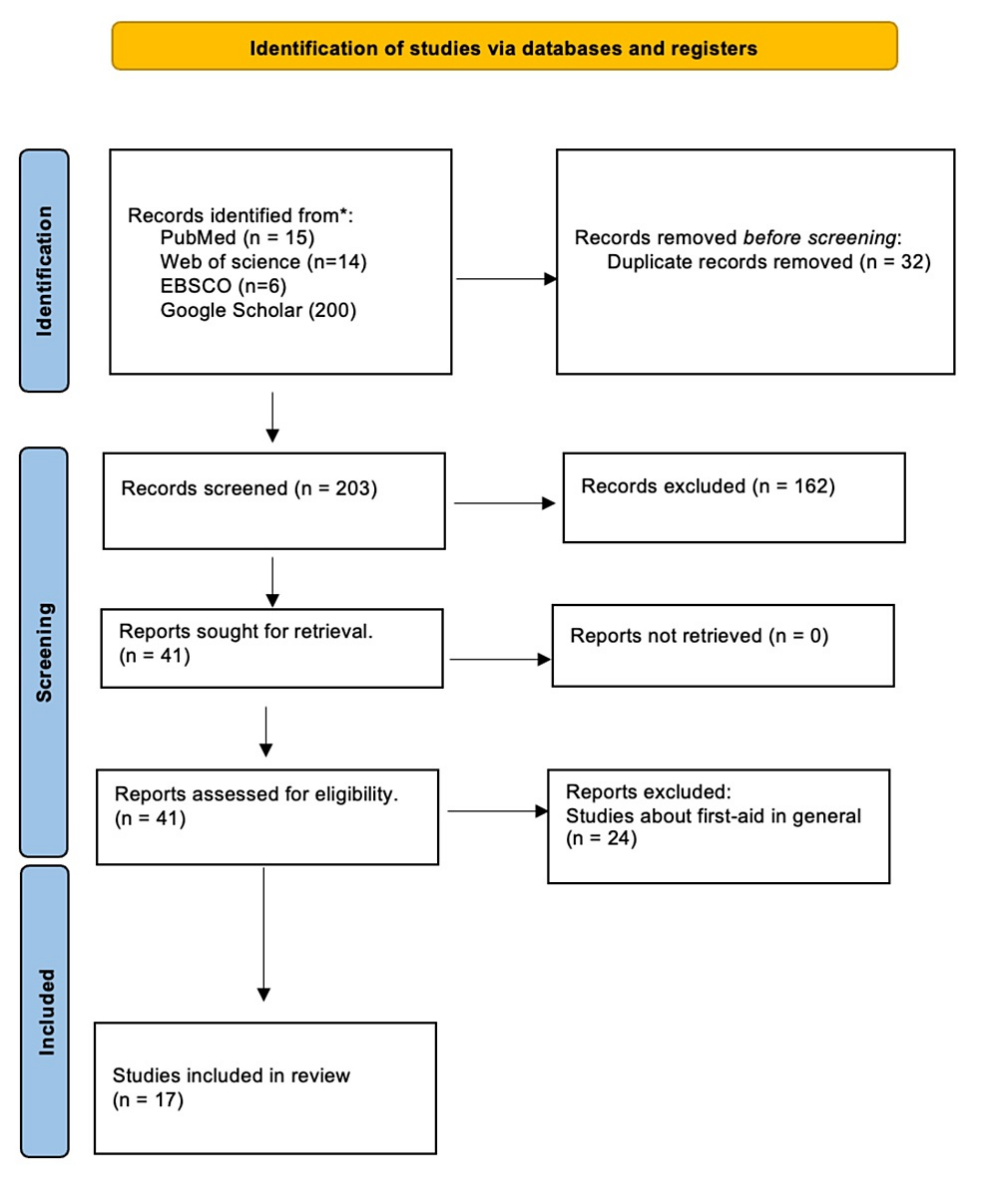
Preferred Reporting Items for Systematic Review and Meta-Analysis (PRISMA) flowchart

The data were independently extracted by four authors (N.I.A., R.K.A., L.A.A., R.H.A.). This included the main author’s name, year of publication, reference number, city, study design, sample size, age, inclusion, exclusion, outcomes, results, and comments. Preferred Reporting Items for Systematic Reviews and Meta-Analyses (PRISMA) were adapted for the methodology statement and data presentation, as illustrated in Figure 1, to conduct the systematic review (SR) and meta-analysis (MA).

Quality Assessment

An Epidemiological Appraisal Instrument (EAI) was to evaluate the quality and score this systematic review via a third party. EAI is an effective tool for assessing the methodological robustness and quality of systematic reviews through a structured framework to evaluate the studies’ designs, conduct, and reporting. “Prior to conducting the ratings, we anonymized all articles by removing titles, author names, and other identifying information. The scoring criteria were as follows: a rating of 'Yes' corresponded to a score of 2, 'No' was assigned a score of 0, and articles labeled 'NA' were excluded from the assessment. To ensure consistency, a consensus on quality assessment was reached among all parties before scoring, enabling the resolution of any disagreements. Subsequently, each study was categorized as either 'High' or 'Low' quality based on the individual scores.”

Results

Database Search

The initial search in the above-mentioned databases yielded a total of 235 studies, from which 32 duplicate records were removed prior to screening. 203 articles were screened and 162 records were eliminated based on the screening text - “Awareness and attitudes toward epistaxis in Saudi Arabia”. A total of 41 articles were then retrieved from the databases and assessed for eligibility based on the above inclusion and exclusion criteria. 24 studies that did not fulfill the selection criteria were removed, leaving a total of 17 articles for inclusion in this systematic review.

Study Characteristics

In Table 1, all 17 studies included for review were of a cross-sectional study design and were carried out in Saudi Arabia. However, they varied widely with respect to geographical area, age, sample size, and inclusion. The variation is illustrated in the table below based on region, age, sample size, and year of publication.

**Table 1:** Characteristics of the included studies CI: confidence interval

Author & year of publication	Region	Sample(n)	Age group	Awareness	Lower 95% CI	Upper 95% CI
Almutairi et al., 2023 [11]	Al Majmaah	407	>15 years	94.0%	0.340	1.211
Al Radhwan et al., 2018 [12]	General Saudi population	600	20-60 year	27.0%	0.110	2.450
Almulhim et al., 2017 [13]	General Saudi population	1114	20 -64 years	67.4%	0.567	2.150
Sahal Arabi et al., 2016 [14]	Al-Madinah	201	>18 years	74.6%	0.654	2.670
Aljuaid et al., 2021 [15]	Taif	377	all ages	80.0%	1.240	6.560
Alhejaily et al., 2019 [16]	Tabuk	540	all ages	45.0%	0.260	1.450
Alanazy et al., 2023 [17]	Qassim region	1152	>15 years	19.4%	0.760	3.450
Merdad et al., 2022 [18]	King Abdul-Aziz University Hospital (KAUH), Jeddah	131	all ages	30.0%	1.140	2.460
Alshehri et al., 2022 [19]	General Saudi population	2441	15 and above	88.0%	0.780	1.930
Alasiri et al., 2022 [20]	Asser Region	394	all ages	76.0%	0.456	1.720
Musleh et al., 2019 [21]	Aseer Region	165	27 -32 years	89.0%	0.246	3.460
Abu‐Zaid et al., 2020 [22]	Jeddah	57	>18 years	72.0%	0.657	1.560
Yassir et al., 2019 [23]	Saudi Arabia	1073	20 - 55 years	33.0%	0.940	2.330
Omar et al.,2020 [24]	Saudi Arabia	1475	>18 years	51.0%	0.770	1.670
Saeed et al., 2020 [25]	Saudi Arabia	400	26-35 years	78.0%	1.020	4.567
Abdullah et al., 2023 [26]	Hail, Saudi Arabia	824	10 – 25 years	63.0%	0.650	2.450
Alshehr et al., 2019 [27]	Jeddah	706	5 and above	79.0%	0.480	3.140

We estimated an odds ratio (OR) comparing the knowledge of respondents regarding epistaxis first aid in Saudi Arabia. Using a Mantel-Haenszel random-effects model, we combined crude ORs. For continuous predictors, we calculated the mean difference and 95% confidence interval (CI) for awareness levels. P denotes the proportion of awareness in the study, and n is the sample size of the study.

Weighted averages of knowledge and awareness proportions were calculated to yield pooled awareness levels for this meta-analysis using the following approach:

Pooled Proportion (P) = ∑ (Proportion of Study * Sample Size of Study) / ∑ (Sample Size of Study)

The standard error of the pooled proportion was calculated in order to determine the lower and upper 95% CI. Z-scores were determined at 1.96 (97.5th percentile of the standard normal distribution) and used to determine the margin of error (MOE) for the 17 studies in this meta-analysis as follows: MOE = Z * SE (Pooled Proportion), whereby Lower CI = Pooled Proportion - MOE, and Upper CI = Pooled Proportion + MOE.

Table 2 provides a quality assessment of the included studies.

**Table 2:** Quality assessment of the studies that were included

Author & year of publication	Selection	Comparability	Outcome	Overall
Almutairi et al., 2023 [11]	3	3	3	Excellent
Al Radhwan et al., 2018 [12]	2	2	2	Good
Almulhim et al., 2017 [13]	3	2	2	Good
Sahal Arabi et al., 2016 [14]	3	2	2	Good
Aljuaid et al., 2021 [15]	3	2	2	Good
Alhejaily et al., 2019 [16]	2	1	1	Fair
Alanazy et al., 2023 [17]	1	1	1	Fair
Merdad et al., 2022 [18]	2	2	2	Good
Alshehri et al., 2022 [19]	3	3	2	Good
Alasiri et al., 2022 [20]	3	2	2	Good
Musleh et al., 2019 [21]	3	2	1	Good
Abu‐Zaid et al., 2020 [22]	3	2	2	Good
Yassir et al., 2019 [23]	2	1	1	Fair
Omar et al.,2020 [24]	2	1	1	Fair
Saeed et al., 2020 [25]	3	2	2	Good
Abdullah et al., 2023 [26]	2	2	1	Fair
Alshehr et al., 2019 [27]	3	3	2	Good

All the articles were published not earlier than October 2017, with three of them being published during the year of publication of this systematic review (2023). There were big disparities in sample sizes, with the largest being 2,441 individuals and the least being 57 individuals. Pooled awareness levels of epistaxis in all the studies under review was 3.76 at a 95% CI of 0.388 - 0.518. The pooled variance of awareness and attitudes toward epistaxis was 1.73652 at a 95% confidence interval. The high aggregated variance across the 17 studies under review indicates the potentiality of heterogeneity - awareness of epistaxis therefore varies widely from one study to the other.

Meta-Analysis

A meta-analysis was conducted to derive combined epistaxis awareness by applying the random effects model, which produces an average estimate across studies, considering their sample sizes. For the purpose of subgroup analysis by age, we categorized participants into three main groups: children (under 18 years), adults (between 18 and 65 years), and the elderly (65 years and older). To summarize the data, we calculated epistaxis awareness proportions, representing the percentage of participants who were aware of epistaxis within the entire study sample. This approach also enabled us to compute the standard error.

Because we anticipated that study outcomes would be heterogeneous, we utilized a random-effects model for analysis. We used the I-square and Cochran-Q statistics to measure heterogeneity and a p-value of 0.1 was regarded as significant. The Beggs regression asymmetry test was used to perform the statistical evaluation for publication bias, and a funnel plot was utilized to show publication bias among the studies used in the meta-analysis.

Forest Plot

**Figure 2:**
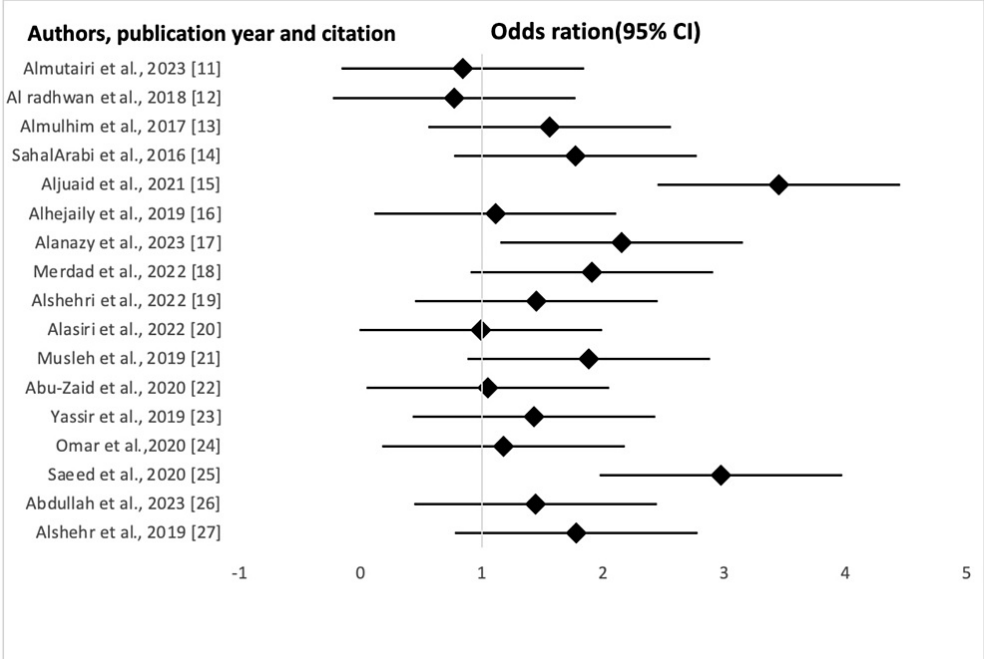
Forest plot depicting the odds ratio

Figure 2 below illustrates the awareness level across the studies. Each study is indicated by the point estimate of the awareness and the 95% CI by random effects.

**Figure 3:**
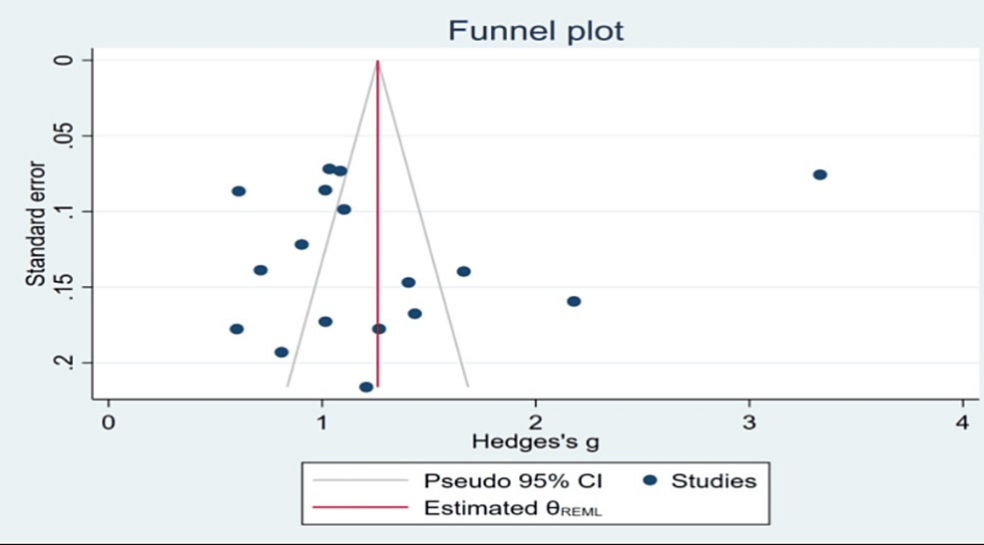
Funnel plot depicting publication bias

Figure 3 illustrates the funnel plot before depicting unequal scatter (asymmetrical), indicating the presence of publication bias.

Discussion

Widespread awareness of epistaxis notwithstanding, the condition is shown to be widely ignored by the general population as a minor health condition and not accorded significant attention [10]. It is imperative to handle epistaxis seriously, owing to the potential consequences of severe bleeding as a medical emergency. This systematic review and meta-analysis presented big disparities in the levels of awareness of epistaxis due to various factors such as the demographic characteristics of the study population like age, gender, geographical location - and statistical variations in sample sizes across the 17 studies reviewed. Knowledge and awareness of epistaxis were observed to be high in studies of the general Saudi population. Awareness was high in studies whose data collection method was mostly self-administered questionnaires. Almutairi et al. demonstrated that Saudi parents who responded to the questionnaire were distributed among the study participants [11]. The majority of studies under review reported moderate awareness and knowledge of first-aid management of epistaxis among the participants, with most reporting that correct positioning of the patient’s head by tilting it forward and applying pressure to control bleeding was the method they were most familiar with. 

Almutairi et al. found that almost all (94%) of the study participants were aware of the proper position that epistaxis patients are required to be in to control bleeding. Sitting with the head tilted forward was used by 48.9% of participants while sitting with the head tilted backward was practiced by 28.7% of participants [11]. Al Radhwan et al. established the lowest awareness level among all studies in this review - most participants (82.3%) didn’t know about epistaxis [12], whereas 74% were not aware of the risk factors associated with the use of certain medicines to address epistaxis. In addition, 66.2% believed that compression of the nose was a risk factor while 68.2% thought it was caused by environmental factors [12].

Almulhim et al. established a relatively high rate of knowledge - about 67.4% of participants were aware of epistaxis first aid management, with self-learning being the most common source of information on managing epistaxis [13]. Sahal Arabi et al. found that 74.6% of the study participants thought of epistaxis as an emergency medical condition mainly caused by a bleeding disorder in the nose [14]. The study also found giving anti-shock treatment, putting the patient in a supine position with the head lowered, compressing the nose, and nasal packing as the most common first aid measures for managing epistaxis [14].

Aljuaid et al. found that most teachers (80.1%) would stop bleeding by applying pressure on the nose, 81.9% responded that they would stop bleeding by altering the position of the head, 49.9% asserted that they would till their head forward and 57.8% claimed that they would put ice on the head or the nose [15]. Only 17% of the teachers claimed that they would try other methods like seeking health care or calling emergency services [15]. Alhejaily et al. found that awareness levels of epistaxis varied across the participants - 34.8% claimed it was caused by chronic diseases, 42.2% stated it was caused by drugs, and 68.9% claimed it was caused by nose manipulation [16].

Alanazy et al. found that levels were so low that just 19.4% of participants had good knowledge [17]. Female gender and prior knowledge of epistaxis management through first aid were significant factors in determining higher levels of awareness [17]. Merdad et al. found that epistaxis awareness levels among health practitioners at King Abdul-Aziz University Hospital (KAUH) in Jeddah were generally poor. Only 30% of the professionals correctly identified the steps of epistaxis first-aid measures [18]. Alshehri et al. found that 69% and 31% of females and males respectively thought that applying pressure on the nose could really stop epistaxis [19], with the upper part of the nose being the most preferred place to press so as to stop the bleeding.

Alasiri et al. found that among the study participants in the Aseer region of KSA, 75.9% of the teachers in this study would apply nasal compression to control epistaxis. When the question of how to pause nose bleeding was posed, 58.1% stated that they would tilt the head forward, and 55.8% claimed that they could put ice on the head or nose [20]. Only 15.5% of the teachers in the study had good awareness regarding the first aid of epistaxis while the rest (84.5%) had poor awareness levels [20]. Musleh et al. found that the majority (88.5%) of the residents knew about the right position of patients with epistaxis. As for the first step to control bleeding, 60.6% of the residents gave the correct answer [21]. Abu‐Zaid et al. found an awareness level of 72% among their study participants [22]. Yassir et al. discovered that of the 403 respondents in their study, 33.3% had good knowledge of its management [23]. Omar et al. established that the overall total knowledge score was good among the population in KSA [24]. Saeed et al. found that knowledge and awareness of epistaxis in the general population was generally good at 78% correctness regarding responses to the questionnaire [25]. Finally, Abdullah et al. and Alshehri et al. established moderately higher, i.e. 63% and 79% awareness levels of epistaxis, respectively [26,27].

The findings of this review were constrained by significant limitations in the studies included for analysis - the lack of a standard method of establishing awareness of epistaxis among the participants as well as variations in sociodemographic characteristics in the samples such as age distribution, gender, region, profession and income levels. Therefore, this review did not consider these factors. Pooling was based on age and gender variations in the studies under review.

A large-scale epidemiological survey of epistaxis is important. Random sampling is recommended for the survey to consider all the above sociodemographic factors. In conclusion, the average awareness and knowledge about epistaxis and how to manage it was 63%, implying relatively moderate awareness levels of the condition among Saudi residents.

## Conclusions

Epistaxis is often overlooked as a minor health concern by the general population. This meta-analysis revealed significant variations in awareness levels across 17 studies, influenced by demographic factors and data collection methods. The average awareness level of epistaxis and its management was approximately 63%, indicating a moderate level of awareness among Saudi residents. While some studies reported good awareness and knowledge of epistaxis management, others indicated lower levels of understanding. Factors like gender, prior knowledge, and profession played roles in determining awareness levels. However, this review faced limitations due to the absence of standardized assessment methods and variations in sociodemographic characteristics. Future large-scale epidemiological surveys, incorporating random sampling and accounting for sociodemographic factors, are needed to obtain a more comprehensive understanding of epistaxis awareness.

## References

[1] Douglas R, Wormald PJ (2007). Update on epistaxis. Curr Opin Otolaryngol Head Neck Surg.

[2] Gottlieb M, Long B (2023). Managing epistaxis. Ann Emerg Med.

[3] Chaaban MR, Zhang D, Resto V, Goodwin JS (2017). Demographic, seasonal, and geographic differences in emergency department visits for epistaxis. Otolaryngol Head Neck Surg.

[4] Faistauer M, Faistauer A, Grossi RS, Roithmann R (2009). Clinical outcome of patients with epistaxis treated with nasal packing after hospital discharge. Braz J Otorhinolaryngol.

[5] Pope LE, Hobbs CG (2005). Epistaxis: an update on current management. Postgrad Med J.

[6] Varshney S, Saxena RK (2005). Epistaxis: a retrospective clinical study. Indian J Otolaryngol Head Neck Surg.

[7] Rockey JG, Anand R (2002). A critical audit of the surgical management of intractable epistaxis using sphenopalatine artery ligation/diathermy. Rhinology.

[8] de Champlain K, Kurek KC, Yunker WK (2018). Novel presentation of cranial fasciitis of the mandible: case report and literature review. Int J Pediatr Otorhinolaryngol.

[9] Welberry C, Macdonald I, McElveen J (2020). Tumor-associated autoantibodies in combination with alpha-fetoprotein for detection of early stage hepatocellular carcinoma. PLoS One.

[10] AlSaleh KA, Al-Numair NS, Alsuaiman A (2021). Prevalence of bleeding symptoms among young adults in Saudi Arabia, a national survey. Medicine (Baltimore).

[11] Alam BS, Jawhari AM, Aljuaid AS, Althomali MA, AlMutairi BS, Alobaylan HA, Alosaimi SM (2023). Parents' knowledge regarding first-aid management of epistaxis in children in Taif, Saudi Arabia. J Family Med Prim Care.

[12] Al Radhwan HM, Alhashim MA, Alkhamis HA (2018). Evaluation of knowledge attitude and practice of general population towards epistaxis in Saudi Arabia. Indo Am J Pharm Sci.

[13] Almulhim KS, Abdulhakim I, Mubarak AS, Hussain MA, Alhaddad MS, Alotaibi NK, Alyahya KA (2017). Assessment of knowledge attitude and practice of epistaxis in Saudi population. Egypt J Hosp Med.

[14] Arabi S, Albouq N, Aljeraisi T, Neyaz H, Alkhurassi H, Alim B (2016). Knowledge and attitude regarding first aid management of epistaxis among medical specialties students in AL-Madinah, Kingdom of Saudi Arabia. Int J Sci Eng Res.

[15] Aljuaid S, Alqahtani RA, Alqasem SH, Alsulaimani YT, Alqahtani SA, Alsalmi SM, Altowairqi RM (2021). Teachers' awareness regarding first-aid management and control of epistaxis inside schools in Taif region, Saudi Arabia. World Fam Med.

[16] Alhejaily MA, Alatawi AA, Alatawi MS, Mrighani HO (2019). Evaluation of knowledge, attitude and practice of epistaxis among the general population of Tabuk City, Saudi Arabia. Egypt J Hosp Med.

[17] Alanazy S, Alqunibut I, Albahli R (2023). The level of school teachers’ knowledge about first-aid management and control of epistaxis in Qassim Region, Saudi Arabia. Cureus.

[18] Merdad M, Sanad SA, Alelyani RH, Alkhammash AM, Swead FM, Alghamdi OM (2022). Assessment of first aid measures to control epistaxis among health care providers at tertiary care hospital in Saudi Arabia. Cureus.

[19] Asiri NS, Alshehri AA, Alshehri FF (2023). Knowledge, attitude and practice of first aid management of epistaxis among general population in Saudi Arabia. Bahrain Medical Bull.

[20] Alasiri AS, Magboul NA, Alasiri AB, Al-Amri D, Albarqi HH, AlAlhareth MS, Alshandari T (2022). Teacher’s awareness regarding epistaxis first-aid management inside schools in Asser Region, Saudi Arabia. Egypt J Otolaryngol.

[21] Musleh A, AlShehri S, AlShehri A, Kadasah S, Alshahrani M, Almuqaytif A, Alsuayri AM (2019). A study of the knowledge and attitude of physicians in the first aid management of epistaxis in Aseer region, Saudi Arabia. Int J Otorhinolaryngol Head Neck Surg.

[22] Abu-Zaid A, Alomari M, AlMazmomy AM, Al-Hayani M, Bazi AG, Althagafi H, Almadani HY (20201). Knowledge of first aid management of epistaxis among medical interns attending King Fahad Armed Forces Hospital in Saudi Arabia. Saudi J Health Sci.

[23] Al-Kubaisy Y, Suwayyid WK, Al-Shakhs AA, Addar LM, Alshammeri MD, Mhraz MY, Alshakhs AM (2019). Teachers’ awareness regarding first-aid management and control of epistaxis inside schools in Riyadh region, Saudi Arabia. International Journal of Medicine in Developing Countries.

[24] Suliman OA, Fallatah EA, Al-Mosa WH, Karsou LS, Al-Junaidy ZZ (2020). Assessment of knowledge, attitude and practice of epistaxis among the population in different regions in Saudi Arabia. Medical Science.

[25] Mohammad S, Alsharidah A, Alshehri M (2020). Knowledge and practice of epistaxis first aid among adult population in Riyadh, Saudi Arabia. International Journal of Medicine in Developing Countries.

[26] Alotaibi AD, Alshammari KF, Alrwaili GF (2023). Do university students have enough knowledge of epistaxis management? A cross sectional study at Hail region in Saudi Arabia. Medical Science.

[27] Alshehri KA, Alqulayti WM, Saggaf OM, Enani MZ, Bahatheq AK, Abdalwassie LK, Marzouki HZ (2019). Awareness of first-aid management of epistaxis among school students in Jeddah, Saudi Arabia. Saudi Surg J.

